# Using document phenomenology to investigate academic failure among year 1 undergraduate Malaysian medical students

**DOI:** 10.1186/s12909-023-04285-2

**Published:** 2023-05-05

**Authors:** Nurul Atira Khairul Anhar Holder, Vinod Pallath, Jamuna Vadivelu, Chan Choong Foong

**Affiliations:** ¹Medical Education and Research Development Unit (MERDU), Faculty of Medicine, Universiti Malaya, Kuala Lumpur, 50603 Malaysia

**Keywords:** Qualitative research, Academic failure, Medical students, Document phenomenology

## Abstract

**Background:**

Academic failure is common among medical schools worldwide. However, the process behind this failure itself is underexplored. A deeper understanding of this phenomenon may avert the vicious cycle of academic failure. Hence, this study investigated the process of academic failure among medical students in Year 1.

**Methods:**

This study employed a document phenomenological approach, which is a systematic process to examine documents, interpret them to attain understanding, and develop empirical knowledge of the phenomenon studied. Using document analysis, interview transcripts and reflective essays of 16 Year 1 medical students who experienced academic failure were analysed. Based on this analysis, codes were developed and further reduced into categories and themes. Thirty categories in eight themes were linked to make sense of the series of events leading to academic failure.

**Results:**

One or more critical incidents commenced during the academic year, which led to possible resulting events. The students had poor attitudes, ineffective learning methods, health problems or stress. Students progressed to mid-year assessments and reacted differently to their results in the assessments. Afterwards, the students tried different types of attempts, and they still failed the end-of-year assessments. The general process of academic failure is illustrated in a diagram describing chronological events.

**Conclusion:**

Academic failure may be explained by a series of events (and consequences) of what students experience and do and how they respond to their experiences. Preventing a preceding event may prevent students from suffering these consequences.

**Supplementary Information:**

The online version contains supplementary material available at 10.1186/s12909-023-04285-2.

## Background

Every year, several medical students are observed to either repeat the year, dropout, or terminate the medical program due to unsatisfactory academic achievement (i.e. academic failure). This scenario results in a decline in the number of students at higher education institutions, which is known as attrition [1]. In a literature review, the average attrition rate for 40 medical schools worldwide was 11.1%, ranging between 2.4% and 26.2% [2]. These findings are worrying and a concern for medical schools worldwide, as attrition rate could be considered as the performance indicator of a medical school [3].

A 30-year retrospective study discovered five possible causes of attrition: personal reasons, transfer to other medical schools, dismissal due to poor academic performance, student death, and unspecified reasons [3]. One of the causes of attrition is poor academic performance in struggling students [4–13]. In this study, a ‘struggling student’ is defined as a medical student who has failed to progress to the next stage of medical study due to poor academic performance in one or all aspects of knowledge, clinical skills, and professionalism [14, 15].

Furthermore, struggling students are at risk of failing to thrive in their medical studies [13, 16]. Students who struggle and fail in the early years of medical studies will continue to suffer the ‘cycle of failure’ until their final year [17, 18]. Moreover, the majority of these students would eventually withdraw or terminate due to the continuation of poor academic performance [19]. Even if struggling students manage to progress, they probably would continue to be weak students and are likely to become incompetent doctors [20, 21]. Incompetency among graduates is at odds with one of the objectives of medical schools and medical professional bodies: to ensure safe and accurate medical care among medical graduates and practitioners [22].

### Literature Review

Previous studies have revealed the possible reasons for academic failure. We have summarised these past findings into four groups: (1) student background (age, gender, prior education level, social class background, ethnicity, and accommodation) [6, 8, 23–27], (2) learners’ issues (poor attitudes, ineffective learning methods, health problems, stress, interpersonal problems, substance abuse, or learning disabilities) [4–13, 28–31], (3) teachers’ issues (personal biases and reluctance to explore student issues) [5, 11, 28, 29], and (4) institutions’ issues (types of curricula) [28–30, 32]. Table 1 summarises these findings according to the possible reasons for academic failure and its associations.

**Table 1:** Possible reasons for academic failure and its associations

Possible reasons for academic failure		Research that showed negative associations. Implying that students are less likely to experience academic failure due to the reasons	Research that showed positive associations. Implying that students are more likely to experience academic failure due to the reasons
**Student backgrounds**	Age	Arulampalam et al., 2004	
	Gender	Arulampalam et al., 2007; Noor, Khan, & Ghazanfar, 2020	Arulampalam et al., 2004; Stetto et al., 2004; *Ford et al., 2008; *Mcloughlin, 2009
	Pre-university academic performance	Arulampalam et al., 2004; Yates & James, 2007	
	Social class	Arulampalam et al., 2007	*Mcloughlin, 2009
	Ethnicity		Yates & James, 2007; *Ford et al., 2008; *Mcloughlin, 2009
	Live outside the campus (accommodation)		Arulampalam et al., 2004; *Mcloughlin, 2009
**Learners’ issues** (e.g., attitude, learning method, health problem, stress or life stressors, interpersonal problem, substance abuse and learning disability)			*Ford et al., 2008; *Mcloughlin, 2009; Evans, Alstead, & Brown, 2010; *Hays, Lawson, & Gray, 2011; Tabby, Majeed, & Schwartzman, 2011; Yates, 2011; Steinert, 2013; Andyryka et al., 2014; *Patel et al., 2015a; *Patel et al., 2015b; Ahmady, Khajeali, Sharifi, & Mirmoghtadaei, 2019; *Ratnapalan & Jarvis, 2020; *Kiran & Javaid, 2020; Ajjawi et al., 2020
**Teachers’ issues** (e.g., personal biases and reluctance to explore student issues)			Evans et al., 2010; Steinert, 2013; Ahmady, Khajeali, Sharifi, & Mirmoghtadaei, 2019; Ajjawi et al., 2020
**Institutions’ issues** (e.g., types of curricula)		Burch et al., 2007	Iputo & Kwizera, 2005; Ahmady, Khajeali, Sharifi, & Mirmoghtadaei, 2019; *Kiran & Javaid, 2020; Ajjawi et al., 2020

Note. *Ford et al., 2008; *Hays et al., 2011; *Mcloughlin, 2009; *Patel et al., 2015a; *Patel et al., 2015b; *Ratnapalan & Jarvis, 2020; Kiran & Javaid, 2020, are the only qualitative studies. Otherwise, other studies were quantitative

Learners’ issues are often reported to have positive associations with academic failure. In other words, students fail because of issues such as poor attitudes, ineffective learning methods, health problems, stress, interpersonal problems, substance abuse, or learning disability [4–13]. First, past studies have revealed discoveries regarding the attitudes of struggling medical students. Struggling students are reluctant to seek help [4] because they often rationalise or downplay their academic failure to cope [10]. Furthermore, some students stopped doing all necessary learning activities as they felt abandoned because of their academic failure, which could worsen things [8]. Struggling students were also observed as ‘dependent individuals’ as they did not ‘take charge’ of their current situation and seemed lacking common sense to rectify issues using common approaches [8]. In addition, poor insight [7], poor self-awareness [6], immaturity [7], poor self-knowledge [8], poor commitment [6], and low motivation [6, 9, 13] were common.

Some struggling students have limited knowledge about the world they live in, as the majority seem to be overly focused on medical knowledge [8] and on the self as a medical student [4]. In addition, most medical students are bright students with excellent pre-university achievements, where they have high expectations of themselves [9] and rarely expect to fail [8]. When these students struggle and fail, it impacts their motivation, emotional well-being, self-esteem, and subsequent commitment. Hence, low mood [9] and poor self-esteem [6] are also common among struggling students.

Second, struggling students adopt ineffective learning methods [6–8, 10], poor organisational skill [6–8], and an inability to adapt to university life [13] due to maladaptive learning methods [6]. Medical students who initially struggle have trouble adopting a new learning method; hence, they continue to use previous learning methods [8]. However, these learning methods are ineffective. Most struggling students have difficulty coping with the academic workload [13] and simply read, read, and read again [8] rather than trying to understand the concepts learned, as it will consume more time and effort. Struggling students also appear to be inept at mentally constructing the ‘big picture’ of the materials learned [4], had difficulty focusing on their study [9], poor time management, inability to adopt regular patterns of study time, poor attendance [6], and poor preparation [8, 12].

Third, heath problems are one of the possible reasons for academic failure [4, 11, 13]. However, these studies did not specify the notion of health problems as they could be either physical or mental health problems. Nevertheless, one study has revealed two types of health problems: medical illness (insomnia) and mental health illness (anxiety and panic attacks) [9]. Depression was also a common issue among struggling medical students [6, 11]. Subsequently, struggling students with poor mental health are resistant to receive remediation [7].

Fourth, students in the medical degree program experienced higher levels of stress than students from other programs [33]. Students normally experience stress related to a medical degree program (i.e. a massive amount of content to study, fast-paced environment, and numerous assessments) and future career concerns [11]. However, when the stress level is too high, it would have negative effects on students’ cognitive functions and subsequent learning experiences [34]. Examples of the negative effects of stress include self-medication, reduced attention during teaching and learning activities, and absenteeism [33]. Studies have also revealed personal life stressors as one of the reasons for academic failure [7, 11]. Examples of life stressors include family issues [13], relationship breakdown, financial hardship, bereavement [9], immigration, marriage, divorce, and moving to another location [11].

Fifth, medical students may experience interpersonal problems. Medical students have many encounters with various groups of people, such as academic staff, non-academic staff, medical peers, non-medical peers, patients, and healthcare professionals. Struggling students are often loners [8], lack social support [4], has poor relationships with patients [5], and interpersonal conflicts with other people [11]. Social isolation and unhelpful attitudes towards peers were common among struggling students [9, 10].

Sixth, approximately 22% of neurology residents have psychiatric and substance abuse problems [12]. About 12.5% of junior doctors misused alcohol, but the way alcohol abuse played a role in struggling and academic failure was not expounded [11]. Seventh and last, a study revealed two examples of learning disabilities, autism and dyslexia, where it possibly impairs students’ learning and contributes to academic failure [6].

Many of the past studies in Table 1 were quantitative studies [5, 11–13, 16, 23–29], where statistics did not explain how and why a student failed. Hence, the process of academic failure is underexplored. Past qualitative studies investigated students of unspecified years of medical study [6–8] or a wide range of undergraduate students from Year 1 to Year 5 [10], or a combination of postgraduate and undergraduate students [9]. We propose that an investigation of the early years of medical study is necessary because of the high attrition rate [35] and dropouts among Year 1 students [19, 23]. Hence, this qualitative study aimed to explore the process of academic failure among Year 1 struggling medical students.

## Methods

### Context

The medical degree program in the medical school at the Universiti Malaya, Malaysia, is an integrated medical curriculum with 5 years duration, which is divided into five stages: Stage 1, Stage 2, Stage 3.1, Stage 3.2, and Stage 3.3. Stage 1 and Stage 2 are equivalent to Year 1 and Year 2 (one-year duration for each stage) and are pre-clinical years. Meanwhile, stages 3.1, 3.2, and 3.3 are the clinical years which are comparable to Year 3, 4, and 5 (one-year duration for each stage).

In this integrated medical program, along with learning the basic knowledge of medicine, medical students also learn the necessary clinical skills (e.g. handwashing, venepuncture, and communication skills) during their pre-clinical years. Students are then able to assimilate and apply what they have learned in lectures (i.e. anatomy, pathology, physiology) to their early patient exposure. Subsequently, during the clinical years, students assimilate and apply what they learned during Stage 1 and Stage 2 into their clinical practice.

In our medical school, the current institutional policy only allows students to repeat the year twice throughout the whole five years of medical program. Students were allowed to repeat once during their pre-clinical years (either in Stage 1 or Stage 2) and another attempt during their clinical years (either in stages 3.1, 3.2, or 3.3). Hence, the maximum time for a student to complete a medical degree is seven years. Therefore, failure in the repeated years of any stage results in immediate termination from the institution.

Stage 1 curriculum is a system-based integrated curriculum consisting of five blocks of systems. At the beginning of the academic year, students were introduced to the Language in Medicine block. Afterwards, they engaged in the remaining four blocks which were the Foundation for Basic and Clinical Sciences, Musculoskeletal Sciences, Cardiovascular Sciences, and Respiratory Sciences. Examples of teaching and learning strategies include didactic lectures, early clinical exposure during clinical days, problem-based learning (PBL), and opportunities for self-directed learning (SDL).

### Data collection

This study was approved by the Universiti Malaya Research Ethics Committee (reference number: UM.TNC2/RC/H&E/UMREC–89). Phenomenology is a qualitative research design used to investigate the lived experiences of individual persons [36]. The phenomenological approach enables university students to express and conceptualise reasons for their academic failures [9, 37]. In the context of this study, the fourth author collected the data (e.g. interviews), but the first author analysed the data (e.g. interview transcripts) to understand the phenomenon of academic failures; hence, it is a document phenomenology. Document phenomenology is a method of describing and interpreting documents to understand investigated phenomena [38]. It is a holistic analysis which considers physical, mental, and social perspectives in the meaning-making process. Document phenomenology has three frames of analysis: (1) documents, (2) parts of documents, and (3) document systems [38].

In this study, the documents used were interview transcripts and reflective essays. The “document systems” may be interpreted as history of the interview transcripts and reflective essays (e.g. who acquired the documents, how and why the documents were collected, what was the context). Documents for 16 struggling Stage 1 medical students from three different cohorts were obtained. The interview transcripts were documents, and six reflective essays (Table 2) were used in tandem with the data from the interview transcripts, generally known as triangulation of data [39, 40]. We use pseudonyms in the Results section to protect anonymity of the struggling students, and the academic years are not reported.

**Table 2:** Details of the data

No.	Student pseudonym	Gender	Ethnicity	Data	
				Interview transcript	Reflective essay
1.	Siti	Female	Malay	20 pages, 455 lines of conversations	1080 words
2.	Devi	Female	Indian	17 pages, 305 lines of conversations	1010 words
3.	Danial	Male	Chinese	17 pages, 292 lines of conversations	956 words
4.	Bong	Male	Chinese	17 pages, 274 lines of conversations	557 words
5.	Tamilseri	Female	Indian	22 pages, 439 lines of conversations	741 words
6.	Ravi	Male	Indian	22 pages, 459 lines of conversations	878 words
7.	Mei Ling	Female	Chinese	21 pages, 511 lines of conversations	-
8.	Se Chan	Male	Chinese	13 pages, 270 lines of conversations	-
9.	Haziq	Male	Malay	11 pages, 178 lines of conversations	-
10.	Ahmad	Male	Malay	11 pages, 213 lines of conversations	-
11.	Lan Fa	Female	Chinese	11 pages, 186 lines of conversations	-
12.	Kavitha	Female	Indian	13 pages, 250 lines of conversations	-
13.	Farid	Male	Malay	10 pages, 242 lines of conversations	-
14.	Zaleha	Female	Malay	12 pages, 356 lines of conversations	-
15.	Letchumi	Female	Indian	12 pages, 314 lines of conversations	-
16.	Nisa	Female	Malay	12 pages, 232 lines of conversations	-

### Data sources

At the institution, the fourth author was the student support person-in-charge, who interviewed individual students who failed Stage 1. This student-support mechanism was offered at our institution. The purpose was to help struggling students to reflect on their experiences and express their emotions. The interviews were audio-recorded and transcribed into Microsoft Word. Some struggling students wrote reflective essays describing their learning experiences. The essays were in the form of Microsoft Word documents. Both interview transcripts and reflective essays were evaluated before documents were used in this study.

#### Evaluating the content in interview transcripts and reflective essays

Interview questions are provided in Appendices A and B. By reading the documents, the first author realised that the students elaborated on what, how, and why it happened. The following excerpt may explain what happened to Mei Ling and how and why she was not able to answer the anatomy assessment.

*“Like last time I didn’t know how to study anatomy because every time I studied anatomy, I just looked at the structure and I memorised the location of the structures and then, but in the exam, in the written exam, the anatomy, what we needed to know was the clinical importance. When this structure becomes problematic, what would be the clinical presentation? This was what came out of the exam. But what I studied was to memorize the location and the movement and, means I focused on the wrong places.”* (Line 330, Interview transcript, Mei Ling)

After evaluating the available documents, the content in both interview transcripts and reflective essays was likely to yield rich data [41]. In addition, the ability to investigate three different cohorts of students can enhance the trustworthiness of the data. These documents were likely to be rich in content and useful.

### Data analysis

Document analysis is defined as a systematic process in examining documents and interpreting them to attain understanding and possibly to develop empirical knowledge [41]. It is a method that combines both content and thematic analyses, where content analysis involves processing the available data according to aims of the research, while thematic analysis involves seeking patterns or themes in the data [41].

There were three phases of document analysis. In the first phase, when reading for the first time, the first author read each document (softcopy) to make sense and familiarise themselves with the data. Subsequently, the documents were re-read (word-by-word) for each student. Every excerpt from the interview transcript and reflective essay was then divided into smaller parts, where each was considered a “meaning unit” [42].

All meaning units were then condensed (if necessary) and subsequently coded, where a code was a label used to capture the idea associated with a segment or the whole meaning unit. Subsequently, all meaning units and codes that were relevant to the research objective were extracted from the original documents (soft copies), and the students’ experiences were arranged in chronological order.

In the second phase, the first author re-read the extracted data. This step was imperative in making sense of the new set of extracted data that were relevant to the research objective, and to review and re-organise students’ learning experiences in a correct chronological order. Subsequently, the process of writing individual students’ learning experiences began. Throughout this process, iterative re-reading (back and forth re-reading process) was performed to make sense of the data and ensure correct interpretation [43].

In the third phase, the write up was re-read. This step was completed with the assistance of a double-monitor computer, where one monitor displayed the written analysis (reading process), whereas the construction and arrangement of relevant codes occurred on another monitor. As a result, the arrangement of codes in chronological order demonstrated the timeline of academic failure for Year 1 struggling students. Three timelines were identified based on the findings: the beginning of the academic year, before and after the mid-year assessments, and end-of-year assessments. One strength of qualitative studies is the ability to trace events over time and explain the reasoning between preceding and succeeding events [44].

**Fig. 1:**
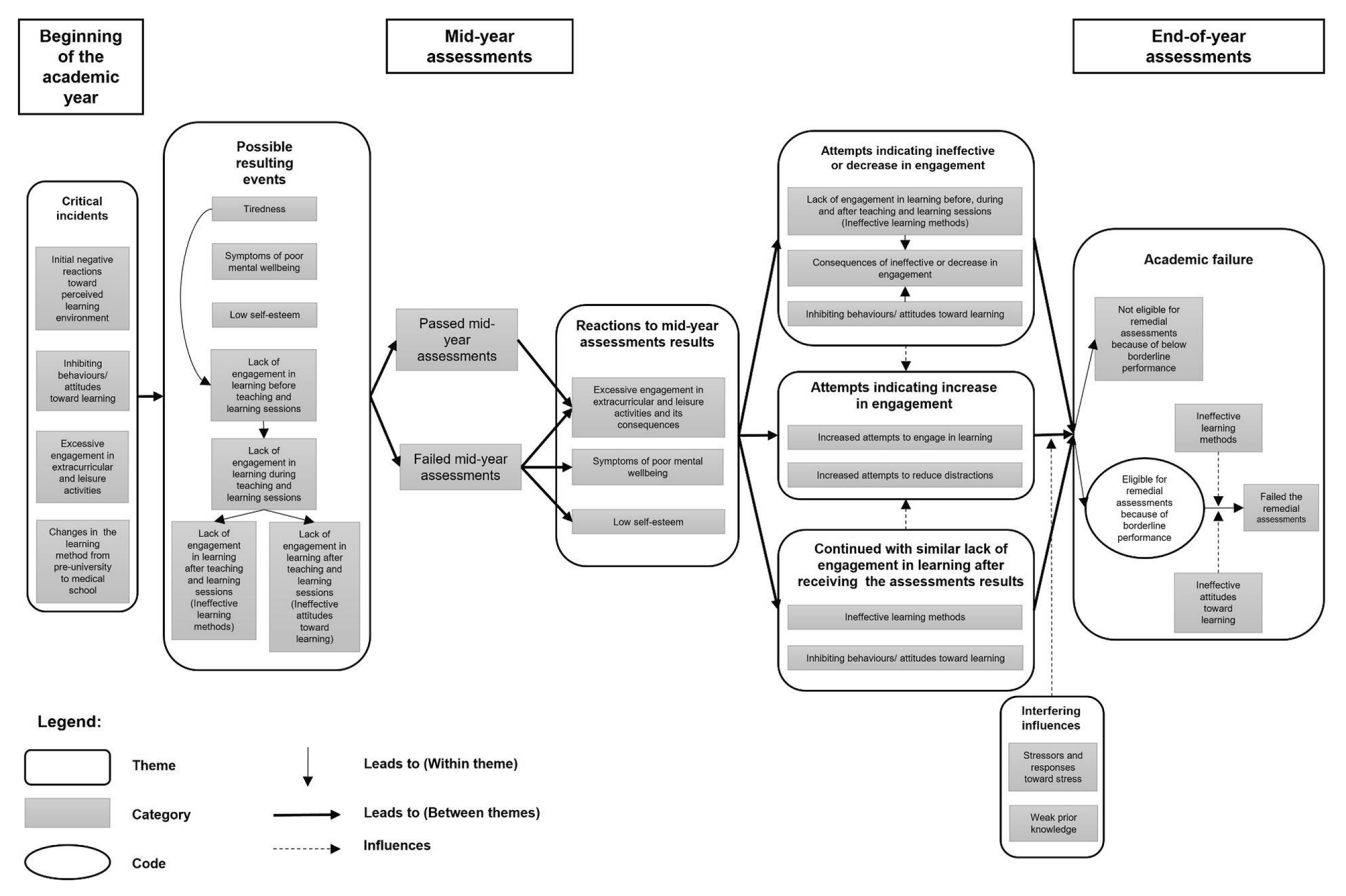
A general model of academic failure

As a result of the three phases of analysis, 222 codes were produced. Next, codes that shared similar characteristics were categorised. An example of a category named “lack of engagement after teaching and learning sessions (ineffective learning methods)” consisted of three codes: did not revise after lectures, did not address poorly understood content, and did not study until the week before the exam. Consequently, 30 categories were identified. Categories that shared similar characteristics were then grouped into themes. An example of a theme named “critical incidents” encompasses four categories: excessive engagement in extracurricular and leisure activities and initial negative reactions towards perceived learning environments. Eight themes were identified. Finally, the categories and themes were linked in chronological order, and the process formed a general model of academic failure (Fig. 1).

## Results

Figure 1 illustrates the general model of academic failure. Themes and categories are described in this section.

### Theme 1: critical incidents

A critical incident is defined as something that a student does well or badly influence the results of their studies. Critical incidents happened at the beginning of the academic year in Stage 1 and was considered as “critical” as the incidents seemed to have initiated the process of academic failure. There are four categories in this theme.

#### Category (i) initial negative reactions toward perceived learning environment

There were several initial negative reactions when the students were engaged in this medical school, which was a new learning environment for them. The Language in Medicine block (first learning block) introduced students to the history of medicine and focused on strengthening their English proficiency. Some students (Siti, Danial, and Lan Fa) felt bored during the Language in Medicine block and found that the first learning block was either useless or relaxed. Meanwhile, Nisa was not expecting to learn English in a medical degree program. When the Foundation block started, some students (Siti, Danial, Ahmad, Farid, and Nisa) found the timetable hectic where they attended lectures from 8am to 5pm, and sometimes until 7pm. Some students also felt that the medical degree program was difficult, and there were many contents to learn.

*“...we started our first two months with Language in Medicine block. Honestly, I was really bored at that time and I did not prepare anything for Foundation block. When the Foundation block started, my life became slightly miserable. The Foundation block was really hectic for me because it was full of classes. Classes started from 8am until 5pm and sometimes until 7 pm.”* (Line 6–10, Reflective essay, Siti)

*“…timetable during Language in Medicine block was quite relaxing. When it came to Foundation block, it was like a sudden turn to a very packed schedule. So, when you have a packed schedule, every day, you have many classes to attend. And every class you have a lot of. So, you have become more stressed. Because you just turned from a relaxing block to a really packed block.”* (Line 238, Interview transcript, Danial)

#### Category (ii) inhibiting behaviours/attitudes toward learning

Inhibiting behaviours/attitudes toward learning was also a critical incident. Some students (Siti, Danial, Farid, and Ravi) were overconfident or lazy about their studies. Their overconfidence or laziness could be due to their excellent academic results. However, this led to other inhibiting behaviours/attitudes, where some students detested studying because of their overconfidence. Meanwhile, other examples of inhibiting behaviours/attitudes, including procrastination, did not take the medical degree program seriously, or did not like to read books.

*“I said just now when I came in, that I was overconfident on myself that I can just finish this in one swipe.”* (Line 271, Interview transcript, Ravi)

*“…I began to despise my studies as I was very confident and sure of passing Stage 1 without breaking a sweat…”* (Line 9–14, Reflective essay, Ravi).

#### Category (iii) excessive engagement in extracurricular and leisure activities

Some struggling students excessively engaged in extracurricular activities. For instance, Siti was involved in sports between colleges, Se Chan participated in basketball competitions, and Ahmad was active in college activities. Ahmad shared those extracurricular activities helped to build on relevant soft skills such as communication skills, leadership, and teamwork. However, he admitted that he did not manage his time properly, as he was carried away with the activities.

*“Because mostly I participated in college activities. I usually develop soft skills, how to interact with people, and how to organise in teamwork. No, I mean how to become a successful leader in teamwork…But the negative part is time management. So, people who want to be active must be able to manage their time properly. I did not manage my time in my case. So I was just, erm, went in the flow…”* (Line 150, Interview transcript, Ahmad)

In addition, some students excessively engaged in leisure activities. This was demonstrated when Ravi often went out to enjoy, play, eat and watch movies. Furthermore, other examples can be seen when Letchumi usually went out or played with her friends, whereas Farid played video games. Farid informed that he turned to playing video games and sleeping to cope with the stress in the medical degree program.

*“During Foundation block, because the timetable was quite packed. Too much to read, so tired. Class packed from morning to evening. Stressful.”* (Line 174, Interview transcript, Farid)

*Interviewer: how did you deal with your stress?*

*Farid: Playing games, sleeping.*

(Line 175 & 176, Interview transcript, Farid)

#### Category (iv) changes in the learning method from pre-university to medical school

Three struggling students changed their learning methods (used previously in their pre-university) after enrolling in medical school. For instance, Siti informed that she read thoroughly and wrote notes during her pre-university education, whereas Devi used mind maps and concept maps instead. Meanwhile, Mei Ling, who used hardcopy notes during her pre-university education, ended up using softcopy notes.

Despite having succeeded in pre-university education with a certain learning method, these students abruptly changed their learning methods upon enrolment in medical school. There was no stated rationale within the interview transcripts regarding the sudden changes in the learning methods of the two students (Siti and Devi). However, Mei Ling justified that her reason for changing the learning method (from using hardcopy to softcopy) was the limited space in her room.

*“Because last time I always read like this. We have used the paper and real notes. Not the softcopies. Then, you just stared at the notes in softcopies, and you just kept scrolling. And then you typed things…”* (Line 212, Interview transcript, Mei Ling)

*“…in the hostel, it’s three person per room. I did not have much space to put my own things. Because my roommates had already come earlier and I was the last one to enter the room, they already occupied all the spaces and the room has not enough space…So if I really wanted to print notes, then there was no space for me to put my notes. Therefore, I decided to not print them. So I just tried to use the softcopies.”* (Line 208, Interview transcript, Mei Ling)

### Theme 2: possible resulting events

Possible resulting events are defined as events that may occur, especially important or unusual, because of critical incidents. There are seven categories of this theme.

#### Category (i) tiredness

Most students were tired, as demonstrated by Siti, Danial, Mei Ling, and Se Chan. Consequently, from one of the critical incidents, some students either woke up late, did not have time to eat and sleep, slept late, had insomnia, or were less prepared for Foundation (Block 2).

For instance, Siti came late or skipped from attending lectures due to oversleeping. When probed deeper, the reason for being overslept was tiredness from participating in the sports competition.

*Siti: What happened? Not really – Actually, I came late to the lecture hall and sometimes skipped lectures.*

Interviewer: why?

Siti: I woke up late. (Laughs)

(Line 38–40, Interviewer transcript, Siti)

*“I think because at the time I was busy with sports competition between colleges. And was tired after the sport practices…”* (Line 50, Interview transcript, Siti)

Meanwhile, tiredness seemed to affect some of the students’ learning, as they did not revise or prepare for the next day’s lectures.

*“…Because attending lectures already take most of the time of the day, and then after that you also participated in activities and then at night, you were tired and didn’t revise or prepare for the lectures…”* (Line 114, Interview transcript, Se Chan)

#### Category (ii) symptoms of poor mental wellbeing

Many struggling students experience symptoms of poor mental well-being (i.e. feeling stress, depression, and panic) following one or more critical incidents. These students (Devi, Danial, Bong, Ahmad, Lan Fa, and Farid) were stressed where some of them also felt miserable or alone.

Several interrelated codes were also observed in some struggling students (e.g. Danial, Bong, and Tamilseri). For instance, Danial was feeling depressed for the first few months in the medical degree program, where he continued to think about why he pursued medicine. Meanwhile, Bong was easily panicked and had issues coping with pressure, whereas Tamilseri experienced cultural shock and was emotionally ‘traumatised’.

*“…When I studied, when I opened the book…but, inside my head, when I will finish this, when will I finish my studies. I was panicked because I always think that I did not have much time…But I always keep that in mind. Theoretically. So I was so panicked when I wanted to cover the lectures in one week.”* (Line 86, Interview transcript, Ahmad)

*“…Because in the first few months I was quite depressed. And always thinking “Why was I taking medicine” Because this course is quite – not to say suffering, but it was hard…”* (Line 16, Interview transcript, Danial)

#### Category (iii) low self-esteem

Some struggling students have low self-esteem. This was portrayed when Tamilseri lost confidence and no longer had an aim because of informal orientation.

*“…After that, I entered Foundation block, and I was so – I don’t know what it is. I lost all my spirit there, and I lost all my confidence there. I started to believe that I cannot…”* (Line 60, Interview transcript, Tamilseri)

*“…I lost the spirit that – in my first year I don’t have an aim. That’s true.”* (Line 276, Interview transcript, Tamilseri).

Meanwhile, Nisa, who reacted negatively to the curriculum in Block 1, was demotivated. Nisa presumed to learn medical content, but in the first three months of medical program, there were lessons of English. She did not feel like a medical student. Mismatches in expectations seemed to have reduced her enthusiasm for medicine.

*“Before I entered the classes, I was like okay; I am going to study medicine. However, when I came, they were English classes. So actually, it has brought all the enthusiasm down. It was almost 3 months. In three months, you like doing nothing in English. In English classes, they said they would teach medical terms. However, we did not actually learn anything from what I’ve learned at school. It is the same, like nouns, and just writes articles, essays, and all. In three months, we did not feel like medical students. So it brought…how I say, because I come with big passion, and I see it English and I feel like…I don’t know what to say but it just went down like that.”* (Line 34, Interview transcript, Nisa)

#### Category (iv) lack of engagement in learning before teaching and learning sessions

Many struggling students lacked engagement in learning before teaching and learning sessions. These students either did not read lecture notes beforehand or printed out the lecture notes in advance but did not read them prior to the lectures. There were also two students who read for the next day lectures; however, they did not read all the lecture notes, as portrayed by Lan Fa and Nisa. Meanwhile, there were two students who differed slightly in their lack of engagement in learning, as evidenced by Devi and Lan Fa. Devi printed out the lecture notes but did not construct her own understanding, whereas Lan Fa read the lecture notes beforehand but did not understand what had been read. In contrast, four students (Siti, Tamilseri, Farid, and Letchumi) skipped attending the lectures.

Although some students were aware of the need to prepare before lectures to ease their understanding of learning new content, they were unable to do so due to time constraints. This was demonstrated by Mei Ling, who spent more time revising lectures on the day than reviewing tomorrow’s lectures.

*“…If you have pre-reading, you will understand more easily…this course is totally new to me, then many terms are not familiar. Then when the lecturers talk, we don’t understand or we do not know the spelling…”* (Line 178, Interview transcript, Mei Ling)

*“Monday got lecture. During Monday nights, I will study Monday morning lectures. Therefore, there was no time to study the Tuesday morning lectures. Means no pre-study. It’s studying afterwards.”* (Line 172, Interview transcript, Mei Ling)

#### Category (v) lack of engagement in learning during teaching and learning sessions

Many struggling students were observed to lack engagement in learning during teaching and learning sessions. These students did not concentrate during lectures, followed by some students who either slept during lectures, participated less in problem-based learning (PBL) group discussions, or simply listened during lectures. Other examples of a lack of engagement in learning during teaching and learning sessions include late for lectures, did not jot down notes during lectures, and did not understand what was being taught during lectures. There was also a situation in which one student (Devi) was unable to concentrate on her learning because of personal issues (i.e. family and financial issues).

It also seemed that the lack of engagement before the teaching and learning sessions resulted in a lack of engagement during the teaching and learning sessions. Many students who did not concentrate during lectures were likely due to no or poor preparation before lectures. Some students also admitted sleeping during lectures, where Tamilseri explained that it was due to tiredness from participating in the informal orientation.

*“During lunch, I have this orientation program and go back and prepare whatever tasks they gave me, and then I have to prepare for a night session. It is likely that they will drag until early in the morning. So I have to skip the classes…even though I didn’t skip for the next class, but I will be going to the class and will be sleeping there…”* (Line 60, Interview transcript, Tamilseri)

Meanwhile, some students seemed to be passive during lectures which was portrayed through the act of passive listening and did not jot down any notes throughout the lectures. Passive learning during lectures seems to indicate that these students were tired of continuous classes.

*“Actually class started at 8 o’clock, (I) will be (a) bit sleepy. And mentally like…if the classes continue, I become so tired. Cannot absorb already. Then, what can I do is listen. I don’t do anything. I don’t take notes or anything.”* (Line 150, Interview transcript, Letchumi)

#### Category (vi) lack of engagement in learning after teaching and learning sessions (ineffective learning methods)

Many struggling students were observed to lack engagement in learning after teaching and learning sessions, specifically with regards to their learning methods. These students did not study until the last week before the examination, did not revise after lectures, did not address poorly understood content, and focused on memorisation. Although some students did try to revise after lectures, they were unable to revise all the lecture notes as evidenced by Siti, Mei Ling, Ahmad, and Nisa. Some students either neglected their studies, relied heavily on lecture notes, or simply read lecture notes without comprehending them. Other examples of a lack of engagement in learning after teaching and learning sessions (concerning students’ learning methods) were disorganised note-keeping and did not refer to other learning references.

Meanwhile, several interrelated codes were observed in some struggling students (e.g. Zaleha, Lan Fa, Letchumi, Tamilseri). For example, Zaleha encountered difficulties while learning when she attempted to address poorly understood content. However, she gave up and learned another topic or subject instead. In Lan Fa’s situation, she forced herself to study despite feeling exhausted. This subsequently had an impact in that she did not understand what had been learned. In addition, Letchumi, who prioritised learning anatomy, ended up procrastinating on learning other subjects, whereas Tamilseri had more unanswered doubts in her study as a result of simply reading the lecture notes without comprehending them.

Many students admitted that they did not revise after lectures in which they did not remember what had been taught previously.

*“Basically after lecture, I just leave it like that. I don’t revise. Yeah. That is why I do not really remember. I don’t know. I don’t study much...”* (Line 118, Interview transcript, Siti)

In addition, some students started studying or revising only when the exam approached. One student (i.e. Ravi) explained that he often studied last minute, and that this learning method worked in his previous education years. Hence, he believed that a similar learning method would still work in medical schools.

*“From my previous experiences when I was in secondary school and matriculation. I would do the same thing you know, starting revision late…So I thought “Okay, if I do that here, I will pass.”* (Line 360, Interview transcript, Ravi)

Furthermore, some students admitted that they did not address the poorly understood content. This was demonstrated by Ravi, who did not make efforts to understand any topic that he struggled with and would simply put the topic aside. Meanwhile, Kavitha, who struggled with her learning, also ignored the difficult content. It seemed that she tried to read multiple times to memorise difficult content, but it did not work.

*“…when I don’t understand something, I didn’t put in enough effort to actually understand that topic. If I don’t understand anything, I will just put it on the side and move on to the next.”* (Line 143, Interview transcript, Ravi)

*“It’s difficult and I just left it to be difficult. I read again and again, in which it did not stick into my head.”* (Line 62, Interview transcript, Kavitha)

Although in some cases, these ineffective learning methods were due to inhibiting behaviours or attitudes and/or excessive engagement in extracurricular and leisure activities, these students did not know how to learn in the context of a medical school.

#### Category (vii) lack of engagement in learning after teaching and learning sessions (ineffective attitudes toward learning)

Some students demonstrated a lack of engagement in learning after the teaching and learning sessions in relation to ineffective attitudes toward learning. For instance, Siti did not spend time studying but instead spent her time on leisure activities (i.e. hiking, watching movies). Danial thought that he could study all medical content once and memorise them. In addition, Danial only studied the content that he deemed important. In Farid’s situation, although he was selective on what to study, he also played games or slept to cope with the medical degree program. Other examples of a lack of engagement in learning after teaching and learning sessions in terms of ineffective attitudes towards learning were studied according to mood and did not have the discipline to manage their own time.

Some students admitted that they studied only when they had mood. It seemed to be a practice that was influenced by their inhibiting behaviours or attitudes. For instance, Danial would study only when he had a mood to do so. If he was not in a mood, he would turn to other activities such as watching dramas or movies. Hence, he was inconsistent in learning because he studied according to his mood.

*“…don’t have the mood to study sometimes. When you do not have a mood to study, you will like trying to relax like watching drama or watching a movie. But when sometimes you have a mood to study like okay, I want to study today, so I will study. So it depends. I do not study every day. It just depends on the mood…”* (Line 182, Interview transcript, Danial)

On the other hand, some students were selective about what to study because of their inhibiting behaviours or attitudes. A student selected a particular lecture note to study according to his own judgement on the level of importance. He rationalised his actions by explaining how tiring it was to study each lecture note, as this would take a few hours. Hence, by being selective, he planned to revise all the remaining unstudied lecture notes when the exam approached.

*“…Just studying a few because studying one note will take a few hours. Therefore, it is sometimes extremely tiring. So, I was just able to pick out a few important notes to study. So, the rest of the notes, just when it comes to the exam. Okay study week, I will study altogether.”* (Line 134, Interview transcript, Danial)

### Theme 3: reactions to mid-year assessments results

Reactions to the mid-year assessment results were defined as students’ responses after receiving their mid-year assessment results. Many students failed the written assessment during mid-year (that is, Siti, Devi, Tamilseri, Ravi, Mei Ling, Haziq, Ahmad, Lan Fa, Kavitha, Zaleha, Letchumi and Nisa), whereas one student (i.e. Danial) failed in the anatomy spot test.

Three students passed all assessments during the mid-year (that is, Bong, Se Chan and Farid). Both Se Chan and Farid were able to revise all the lecture notes. In addition, Se Chan was determined to pass his mid-year assessments, whereas Bong attempted the examination despite feeling anxious and panicked.

The third theme involves struggling students’ responses after receiving their mid-year assessment results. There are three categories in this theme.

#### Category (i) excessive engagement in extracurricular and leisure activities, and its consequences

After receiving the results of their mid-year assessments, regardless of the result of the pass or fail, some struggling students (Tamilseri, Lan Fa, and Se Chan) were excessively engaged in extracurricular and leisure activities (e.g. piano practice for university events, debate competitions, and prayer events). For example, Lan Fa participated in a large event because she liked playing the piano. Every day, she practiced using a piano until midnight. Despite her piano practices, Lan Fa did not forsake her studies. However, her learning method seemed to be ineffective, as she did notes without comprehending what she had written.

*“For the second semester, I joined a very big event…Because I like to play piano…But we practiced until late at night. Each day, every night. Until midnight…And then, I come back, I will not have a nap. After taking a bath, I will just sit down, and I did my own notes…I will force myself to finish my things…I did the notes, but I do not have input. I just did for the sake of doing that...”* (Line 26, Interview transcript, Lan Fa)

In this category, several interrelated codes were observed among some struggling students. For instance, Se Chan was tired of his participation in extracurricular activities. He also watched YouTube videos and went out to socialise with his friends. Tamilseri was involved in competitions after her failure in the mid-year assessments. Although she was excited to be involved in these competitions, it seemed that she regretted wasting her three weeks on not studying. Tamilseri admitted that she did not know how to manage her time. As her time management went off, she continued to oversleep and skipped her lectures.

*“Okay, after the mid-year assessments, I failed, and then we entered Musculoskeletal block…I was quite active with all kinds of competitions, that’s never going to benefit me. However, I was excited to enter it. I think I wasted almost three weeks during the Musculoskeletal block, and then I was like no, no, it should not happen. I have to study.”* (Line 192, Interview transcript, Tamilseri)

*“…But sometimes I will sleep because my time management run away already…In the morning I will sleep. And then I won’t go for the lectures…”* (Line 196, Interview transcript, Tamilseri)

#### Category (ii) symptoms of poor mental wellbeing

Some students experience symptoms of poor mental well-being after receiving the results of their mid-year assessments (Devi and Kavitha). One student described the after-effect of failure in mid-year assessments as an isolation between students who failed and passed their mid-year assessments. For example, Devi felt alone as she felt that her peers were unhelpful.

*“After my mid-year assessments…I had no friends who were helping me around. All my batchmates were… they wanted to study. They had already passed their mid-year assessments. So, it was like quite, an isolation for the – for my other batch mates who had actually failed. So I had to do it all alone…”* (Line 52, Interview transcript, Devi)

Moreover, another student who failed felt more pressured because she felt that she was the only person in her group who failed the assessment.

*“…In mid-year assessment, I failed and all my other friends passed and to be in a group where everyone passed and you alone failed. So horrible failed. So that kind of pressure.”* (Line 170, Interview transcript, Kavitha)

#### Category (iii) low self-esteem

Some struggling students (Devi, Ravi, Kavitha, and Siti) experienced low self-esteem, whereas others were demotivated or lost confidence. Evidence pointed to the impact of low self-esteem, where Ravi lost his confidence and doubted whether medicine was the right course for him. Consequently, he lost the motivation to study.

*“…Eventually I failed my mid-year assessments, and I lost all my confidence.”* (Line 17, Reflective essay, Ravi)

*“…Hm, when I - when I’m in doubt, I don’t have that motivation to actually study. Because you know that, you have that doubt whether I am right from this course or not. So, for me, I just took it as if I am not right for this course, then why should I study? …”* (Line 267, Interview transcript, Ravi)

On the other hand, Siti lost trust in herself and even distrusted her basic knowledge after failing the mid-year assessments. As a result, she had low confidence and seemed to feel insecure, as she felt that her friends comprehended the subject better than her. Subsequently, Siti became worried, stressed, and afraid to participate in problem-based learning (PBL) group discussions.

*“I think low confidence because I do not really trust my basic, basic thing [Note: basic knowledge]. Yeah, everything about my foundation block knowledge. Don’t know why.”* (Line 266, Interview transcript, Siti)

*“Because I can see – because I look at my friends and they look like – they know everything. They understand everything, but I do not. So I worry and be like stressed.”* (Line 268, Interview transcript, Siti)

### Theme 4: attempts indicating ineffective or decrease in engagement

Attempts to indicate ineffective or decreased engagement should be understood as struggling students tried to do something to improve learning; however, these attempts were ineffective or demonstrated a decrease in engagement in studies. There were three categories under Theme 4.

***Category (i) Lack of engagement in learning before, during and after teaching and learning sessions (Ineffective learning methods)***.

Many struggling students lacked engagement in learning before, during, and after teaching learning sessions, specifically in relation to their learning methods. Many students focused on memorisation, relied heavily on lecture notes, and did not concentrate on lectures. For instance, Nisa felt that she understood some of the lecture notes by reading. However, when she proceeded to the next part of the lecture notes, Nisa forgot about the first part that she had already read. Hence, Nisa tried to remember every part of the lecture notes without properly understanding them:

*“…I think because usually after I read, I feel like “Okay, I covered already” and I will go to the next part. But then when I go to the next part, then I forgot the first one…So I just remember whatever in the lecture notes but I don’t remember…like…why is it like that…”* (Line 170, Interview transcript, Nisa)

In this category, several interrelated codes were observed in some struggling students. For instance, Devi did not print out lecture notes and only brought her notebook to jot down content that she ‘felt important’. Afterwards, she returned to her room and constructed her own notes without referring to lecture notes or slides. Meanwhile, after the teaching and learning sessions, Tamilseri did not study until one week before the exam, and she also had disorganised notes. According to Ravi, he only studied up to 60–70% of the content of a topic, and he did not refer to other learning references. In addition, Ravi admitted that he did not know the ‘correct’ learning method.

Next, Se Chan informed him that he had not prepared before the lectures. Se Chan was also unable to focus during lectures where he attributed to its long duration of time. As Se Chan lost his focus during the lectures, he did not jot down notes and, consequently, did not remember what was taught during lectures.

*“…Maybe the lecture time is too long. And yeah, somehow you misfocus.”* (Line 46, Interview transcript, Se Chan)

*“Maybe the lecturer says some important points I didn’t jot down. I didn’t remember it…”* (Line 56, Interview transcript, Se Chan)

After the teaching and learning sessions, he did not revise most of the time. If he did revise, Se Chan would simply read the lecture notes without comprehending them and would not refer to any reference textbooks.

Other examples of lack of engagement in learning before, during, and after teaching and learning sessions are skipped from attending lectures, not preparing for PBL group discussions, slept during lectures, forced self to study for the sake of doing, and studied everything generally.

#### Category (ii) inhibiting behaviours/attitudes toward learning

Some students (Tamilseri, Se Chan, Siti, and Danial) exhibited inhibiting behaviours or attitudes toward learning. For example, they did not carry out the planned changes to completion, were not disciplined in managing their own time, and emphasised an assessment over other assessments, even though they were required to pass all the assessments.

For instance, Se Chan was not disciplined in managing his time to study. Even though he had plenty of time to study before his study week (one week before the assessments), his poor time management and last-minute study resulted in him not having enough time to study.

*“No, I mean is before the study week, I have plenty of time…But sometimes at events and sometimes I didn’t have proper time management in study. So it leads me to feel that I don’t have enough time to study.”* (Line 220, Interview transcript, Se Chan)

Meanwhile, Danial had difficulty prioritising his weak subject (anatomy), where he focused more on other assessments instead of anatomy.

*“…But there’s – the first time I failed for end-of-year assessment was because I gave up the Anatomy Spot Test before the end-of-year assessment. Therefore, I gave up on the opportunity to study anatomy. I consider that–I concentrate on my end-of-year assessment. So that’s why – maybe the reason I failed for the first time, for the Anatomy Spot Test…”* (Line 40, Interview transcript, Danial)

On the other hand, Tamilseri was observed to have more than one inhibiting behaviour or attitude toward learning. Tamilseri admitted that she was lazy and procrastinated in her study. She also did not follow through with her planned changes and expected spoon-fed teachings.

#### Category (iii) Consequences of ineffective or decreased in engagement

Consequences of ineffective or decreased engagement in learning have been observed in many struggling students. Some students did not have enough time to revise all contents within the Year 1 medical degree program. In addition, these students did not understand what they had learned when they were unable to link the different topics taught.

*“I do think. I memorize everything. I do not understand this fully. In medicine, one cannot memorise things. You have to do some connections between this and this.”* (Line 70, Interview transcript, Letchumi)

*“…And I don’t really know how to interrelate them between the subjects…actually have to link together so that we can understand the concept well. Therefore, if the question comes out in (a) different way, we can still answer. It is not like an answer directly. I mean yeah because the end-of-year assessments’ questions tests our understanding of the concept of basic science. So I think, maybe this, the understanding aspect, I didn’t do quite well.”* (Line 28, Interview transcript, Se Chan)

Meanwhile, other examples of the consequences of ineffective or decreased engagement in learning were portrayed by Mei Ling, who did not understand what she memorised, and Danial, who gave up studying the subject Anatomy.

### Theme 5: attempts indicating increase in engagement

Attempts indicating increase in engagement should be understood as struggling students trying to do something to increase their engagement in studies. There are only two categories of this theme.

#### Category (i) increased attempts to engage in learning

After receiving the mid-year assessment results, many struggling students increased their attempts or attempted several changes to engage in learning. Many students were observed to increase their study time after failure in mid-year assessments.

*“Before that, I liked to study at night. At least one hour. However, after the mid-year assessments, I studied for more than four hours. I will start studying from 7pm or 8pm until 12 midnight.”* (Line 82, Interview transcript, Letchumi)

Some students tried to modify their learning method by writing short notes, reading notes before the next day lectures, staying awake during lectures, and revising after lectures. Meanwhile, some students also sought advice from other people (i.e. seniors and academic advisors), were involved in a study group, or tried to study with friends.

*“I do my short notes, but at that time I don’t read it repeatedly. It’s like, I will do, and then I will wait for the last minute, like that. That was the mistake that I’ve made.”* (Line 222, Interview transcript, Tamilseri)

*“…And after I failed my mid-year assessment, I went around and start asking seniors on how to study, how to improve, how to make sure I know…”* (Line 26, Interview transcript, Kavitha)

#### Category (ii) increased attempts to reduce distractions

Some students (Siti and Ravi) attempted to reduce distractions. This was demonstrated by reducing leisure activities and switching off phones during lectures.

*“As I said earlier, I limited my time for going out and spent more time on studying. If I studied, let’s say, for mid-year assessments, I studied for two hours. Then, for end-of-year assessments, I made them for four hours. Then during lectures, I would actually switch off my phone so that I won’t get distracted by incoming WhatsApp messages and all that stuff…”* (Line 171, Interview transcript, Ravi)

### Theme 6: continued with similar lack of engagement in learning after receiving the assessments results

Continued with a similar lack of engagement in learning after receiving the assessments’ results should be understood as struggling students were continuing to do the same thing as before their mid-year assessments, despite their failure. There are two categories in this theme.

#### Category (i) ineffective learning methods

Many struggling students continued with similar learning methods as before the mid-year assessments. Some students did not read lecture notes beforehand, did not understand what they had learned, skipped lectures, and did not have enough time to revise all contents.

*“…I didn’t study before that. I only attend lectures, jotting down and in the evening, I will recall back.”* (Line 80, Interview transcript, Kavitha)

Meanwhile, some students also admitted that they procrastinated in their learning and preferred to study last minute instead.

*“…But when I come here, I started to procrastinate. I will just do the short notes, keep them aside, and then go to another thing. However, I will not come back and read for that. I will wait for the last – until the last minute to study…”* (Line 230, Interview transcript, Tamilseri)

In addition, several interrelated codes were observed in one student in this category. For instance, Farid did not revise after the lectures. He also did not study until one week prior to the exam and focused on memorisation.

#### Category (ii) inhibiting behaviours/attitudes toward learning

Some students continued with similar inhibiting behaviours/attitudes toward learning. For example, Siti ignored what she did not understand, and Ravi did not address the content that he poorly understood. Meanwhile, Farid continued to be selective regarding what to study.

*“Oh, actually I cover for the major subjects like anatomy, physiology, pharmacology. Like minor subjects like immunology, respiratory, macro… so that minor subjects, I do not need to read. Because many questions come from major subjects such as anatomy… The questions come from that subject. Therefore, I think I just need to cover the major subjects. The minor subjects, I think I don’t need to read because the answer is there, I just need to choose.”* (Line 66, Interview transcript, Farid)

### Theme 7: interfering influences

Interfering influences are events that interfere with students’ positive efforts to increase their engagement in learning. There are two categories in this theme.

#### Category (i) stressors and responses towards stress

Many students were stressed (that is Siti, Haziq, Devi, Letchumi, Ahmad, Kavitha, and Zaleha) where most of the stresses were due to abundance of content to learn within the medical degree program. Each student responded differently to the stress. For example, Siti went out, watched movies, and hiked whenever she was stressed, whereas Ahmad took time to cope with stress and his failure in mid-year assessments.

In this category, several interrelated codes were observed among some students. For instance, Devi was stressed because she was uncertain about the effectiveness of her new learning method. Hence, she jogged or listened to music to cope with the stress. On the other hand, Zaleha was stressed, as her roommate was able to understand with little effort. She was also stressed because of the huge amount of learning content she did not understand. Consequently, she became emotionally unstable and wanted to change to another program one month prior to her end-of-year assessments. Fortunately, her morale improved after meeting with the counsellor.

*“I realise it a month before the end-of year assessments. I have the urge to change my course.”* (Line 108, Interview transcript, Zaleha)

*“What drive…maybe because of the things that I don’t understand, It’s many and becomes like a mountain, and I feel very stressed and I feel like to change my course.”* (Line 114, Interview transcript, Zaleha)

#### Category (ii) weak prior knowledge

Some struggling students might have failed because of their weak prior knowledge (Haziq, Siti, Ravi, and Ahmad). Since the Foundation block taught basic knowledge, it became difficult for them to understand what was going to be taught in subsequent blocks.

*“…I do not really know what I’ve learned for foundation block. This means that my basic knowledge is weak. Too bad for me, I do not study hard, even though I know that I do not really understand basic knowledge. I am too relaxed and in my comfort zone...”* (Line 14, 16–18, Reflection essay, Siti)

*“Because when ones don’t have the basic knowledge, it can lead to negative effect on subjects that are related to it. For example, your basic knowledge is not strong in relation to the physiology. So you do not understand terms that have already been used in physiology. Therefore, it is more difficult to understand the next block. That’s why.”* (Line 168, Interview transcript, Ahmad)

### Theme 8: academic failure

Academic failure is defined as the endpoint at which students failed their end-of-year and/or remedial assessments. There are four categories in this theme.

#### Category (i) not eligible for remedial assessments because of poor performance

Many students were ineligible for remedial assessments because of their borderline performance during the end-of-year assessments. Ten students failed their written assessments during the end-of-year assessments. In contrast, only one student (Bong) failed all his end-of-year assessments due to absenteeism.

All students were required to repeat Year 1. It also seemed that the borderline performances in the end-of-year assessments were a surprise for most struggling students.

*“I did not realize. I like studying and studying. I knew that the effort was there. I knew I did not play the fool, as others did. I thought I would get the chance to sit for a remedial assessment. I thought if worst comes to worst; it’s going to be remedial assessment, not a failure.”* (Line 140, Interview transcript, Kavitha)

*“So when I failed, it was very unexpected for me. I thought I will pass. At least, I will be able to sit for a remedial assessment. But I totally failed. So it’s like an unexpected thing for me…”* (Line 276, Interview transcript, Tamilseri)

#### Category (ii) eligible for remedial assessments because of borderline performance

Five struggling students were eligible for remedial assessments, but all of them failed the remedial assessments. Mei Ling, Haziq, Lan Fa and Farid failed in the written remedial assessments, whereas Danial who did not know how to answer during exam failed in his remedial Anatomy Spot test. Subsequently, they were required to repeat Year 1.

Some students also seemed unable to accept their failure, as they used to excel in their previous examinations.

*“…Erm, for the first time I know I failed, I will go for remedial assessment, but still, I cannot accept. Because this is my first failure in life, I can say. Before this, I always did very well in my exams. And then I seriously I don’t know why…”* (Line 38, Interview transcript, Lan Fa)

#### Category (iii) ineffective learning methods

All five struggling students who were eligible for remedial assessments continued to use ineffective learning methods. Some students read lecture notes without even comprehending them as they focused on memorisation. As a result, they could not make connections between topics or subjects.

*“…I study but I did not fully understand the topic.”* (Line 68, Interview transcript, Letchumi)

*“…I memorise everything. I do not understand this fully. In medicine, one cannot memorise things. You have to do some connections between this and that.”* (Line 70, Interview transcript, Letchumi)

It seemed that the learning methods were employed, as these students did not have enough time to revise all contents, and they also did not prepare for the remedial classes. In addition, some students reported that they learned the appropriate learning method only during remedial classes. For instance, lecturers taught Lan Fa to think logically about the adverse effects of drugs instead of memorisation. This was in contrast with her previous learning experiences, in which she had memorised. However, even though she was aware of the benefits of thinking, Lan Fa thought that thinking processes were energy-exhausting.

*“During the remedial class, lecturers train us, like for pharmacology, for certain drugs that have these adverse effects. Therefore, they will let us trigger ourselves to think about adverse effects. Not by memorizing. Before that, I memorised the adverse effects of the drugs. However, during the remedial class, the lecturer will train us to think about the drug. The mechanisms of the drugs we will think for ourselves about their adverse effects. I think I will use this approach. Use more thinking than memorising. However, thinking is sometimes exhausting. Very tired thinking a lot.”* (Line 168, Interview transcript, Lan Fa)

In this category, several interrelated codes were observed among some students. For instance, Danial was absent from most remedial classes and studied to memorise everything for his remedial Anatomy Spot test. Meanwhile, Farid simply read lecture notes without comprehending them, similar to Danial, who focused on memorisation.

#### Category (iv) ineffective attitudes toward learning

Some students (Danial, Mei Ling, and Haziq) continued to have ineffective attitudes toward learning, despite being eligible for remedial assessments. For instance, Danial was confident in his own ability to memorise and ended up underestimating the remedial Anatomy Spot test.

*“I think…my own self problem, I think. This is still an overconfidence. Underestimate the paper. And everything you think can memorise, then it means the paper will goes well. Maybe that’s overconfidence also...”* (Line 146, Interview transcript, Danial)

Meanwhile, Mei Ling and Haziq expected spoon-fed teaching during their remedial classes.

*“Because at that moment if, at that moment for me, during the remedial class, we didn’t have the time to know everything. So if we have a question like, sample questions, we will get like, oh this type of sub topics they can ask like this.”* (Line 72, Interview transcript, Haziq)

*“For me, I didn’t like the way the lecturers just go through and didn’t point out the important things…during the remedial class…So during the class I don’t think I got - I didn’t get anything.”* (Line 74, Interview transcript, Haziq)

## Discussion

Past studies have revealed seven issues related to learners (poor attitudes, ineffective learning methods, health problems, stress, interpersonal problems, substance abuse, and learning disabilities) [4–13]. In this section, we compare our findings with those of previous studies. First, regarding poor attitudes, our findings echo past studies that found a link between poor attitudes and academic failure [6–8]. Students with poor insight are difficult to remediate [7]. Based on Fig. 1, the links are such as “Inhibiting behaviours/attitudes toward learning (Critical incident) → Lack of engagement in learning before, during and after teaching and learning sessions (Possible resulting events) → Failed in the mid-year assessments” and “Reactions to mid-year assessments results → Inhibiting behaviours/attitudes toward learning (Continued with similar lack of engagement in learning after receiving the assessments results) → Academic failure”. As many past studies have been quantitative in nature, our study attempted to explain the link between how and why these poor attitudes lead to academic failure.

In addition, in this study, the students seemed to lack conscientiousness despite not using the term conscientious, they repeatedly demonstrated inhibiting behaviours/attitudes towards learning. On the other hand, conscientious students analyse and weigh their situations and subsequently respond to situational demands by adjusting their learning methods [45–47]. Conscientiousness was found to be a positive predictor of performance during the pre-clinical years [6].

Second, regarding ineffective learning methods, our findings align with those of the previous studies. The literature pointed out several issues pertaining to learning methods, such as poor organisational skills [6–8], difficulties in focusing on studies [9], poor time management, inability to adopt a regular study pattern, poor attendance [6], and poor preparation for learning [8, 12], as reasons for academic failure. These reasons were observed in the present study. In addition, we enrich the existing body of knowledge by describing how lack of engagement before teaching and learning sessions would have negative consequences during the sessions and how it further influenced their engagement in studies after the sessions took place. For example, some students who did not read lecture notes beforehand might not be able to concentrate during lectures, slept during lectures, or did not understand what was being taught during lectures; furthermore, after the lectures, they did not address poorly understood content or focus on memorising the content.

Third, regarding health problems, the literature revealed health problems such as medical illnesses (e.g. insomnia) and poor mental health illnesses such as depression [12], anxiety, and panic attacks [9]. Correspondingly, our study discovered the symptoms of poor mental well-being and insomnia. Additionally, our study revealed a link between health problems and academic failure. Based on Fig. 1, the links are such as “Critical incident → Symptoms of poor mental wellbeing (Possible resulting events) → Failed in the mid-year assessments” and “Symptoms of poor mental wellbeing (Reactions to mid-year assessments results) → Continued with similar lack of engagement in learning after receiving the assessments results → Academic failure”.

Fourth, regarding stress in studying medicine, our findings support the literature that medical students experience stress related to medical degree programs [11], family issues [13], and financial hardships [9]. In our study, stress was observed before the end-of-year assessments. Some struggling students were stressed because they had too much content to learn, experienced failure in mid-year assessments, employed new learning methods, or had a roommate who was able to comprehend topics with less effort. Our findings also demonstrated their differing responses to stress, where some were emotional and wanted to change course, while others engaged in activities such as jogging, listening to music, going out, watching movies, and hiking. On the other hand, stressors and student responses toward stress also interfered with struggling students who attempted to increase their engagement in learning.

Fifth, regarding interpersonal problems, although the literature described interpersonal problems as loners [8], lacking social support [4], having poor relationships with patients [5], presenting with unprofessional behaviour [4], or having interpersonal conflicts with other people [11], our study did not have adequate evidence. Similarly, regarding substance abuse and learning disabilities, our data did not register such findings despite a few studies relating substance abuse (i.e. alcohol misuse) [11, 12] and learning disabilities (e.g. autism and dyslexia) [6, 11] to academic failure. In our study, learning disabilities among medical students may have been under-diagnosed because they are always perceived as smart students [48]. Regarding substance abuse, there is a possibility that Asian students may be reticent about substance abuse, possibly due to cultural stigma [49]. Malaysian society perceives drug abuse as unacceptable due to reasons such as criminalisation of drug use in Malaysia [50] and stigmatised conditions related to drug abuse such as HIV [51]. On the other hand, there was also stigma related to alcohol in the Malaysian Muslim-majority society. This may explain why substance abuse was not a problem leading to academic failure in our study.

Our findings have implications for future practices aimed at minimising or remediating struggling students. In our study, a series of events leading to academic failure were revealed, which implies that struggling students may intervene at different points in time to prevent failures. Prevention is best when critical incidents occur at the beginning of an academic year. First, the medical school can inform students about the expectations of learning medicine upon their enrolment. Setting early expectations (e.g. informing the realities of studying medicine) could impede potentially difficult situations [11]. Second, medical students at early stages of medical degree programs are observed to be struggling with knowledge, ways of thinking, and learning methods [52]. In Malaysia, secondary school education has focused on teacher-centred and spoon-feeding [53]. In comparison, adult learning necessitates self-regulated learning which is characterised by students taking responsibility for and control of their own learning [54]. Hence, medical schools can assist their students in transitioning from passive to engaged and proactive learners [55].

Third, some struggling students repeatedly demonstrate behaviours and attitudes that lead to academic failure. Struggling students tend to assess themselves inaccurately (e.g. unaware of their own weaknesses or limitations) which could result in overestimation of self [52] and worsen their issues. Hence, there is a need for remediation that emphasises reflection and supports students in being aware of what they know and do not know, subsequently creating a personal action plan to address them [55]. Finally, effective remediation goes beyond remediating struggling medical students. It is also about facilitating students to explore alternative career pathways that best suit their abilities and personalities [52]. Hence, career counselling may be considered for struggling students to re-align the direction of their future careers [56].

### Strengths and Limitations of the study

A number of qualitative studies were conducted in Europe [6, 7, 9, 10]. There is a possibility of differing student perspectives or experiences due to cultural differences (e.g. in terms of thinking, learning patterns, and behaviour) [57], where the reasons for academic failure and coping strategies could differ in the Malaysian-Asian population.

Our study has several strengths. Continuous comparisons between the extracted data and original documents were conducted during data extraction and analysis to verify the accuracy of the data [58]. Rich and thick verbatim extracts [59] from each struggling student were also utilised, with clear reference to the source of data. Moreover, the procedures of data collection, data analysis, and reporting of findings have been described in detail [59] to allow exact replication for future research. In addition, audit trails of the research process [58] were documented. Details of the context (i.e. medical degree program, assessments and examination regulations, and participants) were also elaborated with the purpose of providing rich and thick descriptions [59], which facilitates readers to evaluate the findings and conclusions from our study and its applicability to other universities or degree programs [39].

Our study has some limitations. First, we were unable to determine the motives behind why some struggling students did what they did. For instance, why did they repeatedly demonstrate the same behaviours or attitudes that initially led to academic failure? This may be due to the lack of self-reflection among students, but this warrants further investigation. Second, our data were based on struggling students’ self-reported data about their perceptions, thoughts, and feelings. The self-reported data were taken as truthful representations of reality.

## Conclusion

Academic failure is a complex phenomenon in which there are reasons interplaying. Academic failure may be explained by a series of events (and consequences) of what students experience and do and how they respond to their experiences. Preventing a preceding event may prevent students from suffering these consequences.

## Electronic supplementary material

Below is the link to the electronic supplementary material.

Supplementary Material 1

## Data Availability

The datasets collected and analysed during this study are available from the corresponding author upon reasonable request.
