# Correction: Using document phenomenology to investigate academic failure among year 1 undergraduate Malaysian medical students

**DOI:** 10.1186/s12909-023-04400-3

**Published:** 2023-06-05

**Authors:** Nurul Atira Khairul Anhar Holder, Vinod Pallath, Jamuna Vadivelu, Chan Choong Foong

**Affiliations:** Medical Education and Research Development Unit (MERDU), Faculty of Medicine, Universiti Malaya, Kuala Lumpur, 50603 Malaysia

**Correction: BMC Medical Education (2023) 23:310**

10.1186/s12909-023-04285-2

Following publication of the original article [1], we have been informed that Reference [25] is incorrectly written.

The original article has been corrected.
