# Supplementary material for: Using document phenomenology to investigate academic failure among year 1 undergraduate Malaysian medical students
Source: BMC Med Educ. 2023 May 5;23:310. doi: 10.1186/s12909-023-04285-2 (PMC10161666; doi:10.1186/s12909-023-04285-2)
Supplement: Supplementary file 1 — Supplementary Material 1 [file 12909_2023_4285_MOESM1_ESM.docx]

**Appendix A: Questions asked during semi-structured interviews.**

Reasons for studying medicine

1. Could you share with me, why did you choose to study medicine?

2. When did you realise that you wanted to become a doctor?

3. Your pre-university results were excellent; why did you not consider choosing other courses?

4. What was your image of medicine before you entered medical school? As compared to this image, how different or similar is it to the actual image (now)?

Possible reasons for academic failure

1. Did you ever feel that you were going to fail before the final exam?

2. When did you realise that you might fail?

3. What did you do when you realised that you were going to fail?

4. Now that you have failed, what has happened?

5. What factors may have contributed to your poor achievements?

- How did the (particular) aspect [ask each at a time] influence your studies?

7. When you failed the main exam, you attended the remediation. How did you feel about the remediation?

8. How did you study?

- What was your learning and studying style?

9. How was your attendance at the lectures?

10. You completed continuous assessments before the final exam; did anyone give feedback based on the assessment results?

- What did they tell you? [If students answer Yes]

11. It is known that studying medicine is stressful. On a scale from 0 to 5, how would you rank your stress?

12. Are you aware of student supports/counselling in this medical school?

- Why did you not seek help from medical school? [If students answer Yes]
- How did you cope with your stress or problems?

15. Based on your experience, how would you advise your juniors so that they do not fail the exam?

16. I know it is impossible now, but what can the medical school do at that time to help you?

17. Some repeating students say that people look at them differently; what is your personal experience?

- When you told your parents that you had to repeat it, what did they say?
- What do your friends say when they know that you have failed the exam?

18. Given a scale from 0 to 5, how confident do you pass the exam this repeating year?

**Appendix B: A list of possible reasons for academic failures asked at the end of the interviews.**

1. I will go through a list with you, whether you think this is the reason for your failure. Some students said that they could not master the medical content because they were weak in English [each point will be replaced with a list below]. What was your experience with it?

- Peer pressure
- Friends’ influence
- Family matters
- Personal problem
- Financial problem
- Health problems
- Language barrier
- Religion or culture issue
- Lack of motivation to study medicine
- Lack of confidence or over-confidence
- Lack of discipline
- Poor time management
- Procrastination
- Learning difficulties
- Prior knowledge (weak in Biology)
- Issue with medical teachers/institution
- Lack of direction or feedback on performance
